# Clinical and laboratory parameters associated with bloodstream infections among neonates admitted to a tertiary hospital in Tanzania: a hospital-based cross-sectional study

**DOI:** 10.3389/fped.2026.1778065

**Published:** 2026-04-20

**Authors:** Delfina R. Msanga, Farida I. Mkassy, Oliver Kurzai, Christoph Härtel, Stephen E. Mshana

**Affiliations:** 1 Department of Pediatrics and Child Health, Weill Bugando School of Medicine, Catholic University of Health and Allied Sciences, Mwanza, Tanzania; 2 Department of Epidemiology, Biostatistics and Behavioral Sciences, Weill Bugando School of Medicine, Catholic University of Health and Allied Sciences, Mwanza, Tanzania; 3 Institute for Hygiene and Microbiology, University of Würzburg, Wuerzburg, Germany; 4 Department of Pediatric and Child Health, University of Würzburg, Wuerzburg, Germany; 5 Department of Microbiology and Immunology, Weill Bugando School of Medicine, Catholic University of Health and Allied Sciences, Mwanza, Tanzania

**Keywords:** C-reactive protein, gram negative bloodstream infection, low-resource settings, neonates, sepsis

## Abstract

**Background:**

Timely diagnosis and effective management are critical for improving outcomes in neonatal bloodstream infections (BSIs), particularly in resource-limited settings. This study aimed to determine clinical signs and laboratory markers associated with BSIs among neonates at Tertiary hospital in Mwanza, Tanzania.

**Methods:**

A hospital-based cross-sectional study was conducted, among neonates treated for suspected BSI from January 2023 to June 2024. Clinical signs of sepsis were systematically documented. Blood samples were collected for culture, serial C-reactive protein (CRP), and complete blood count at admission, 24 h, and 72 h. Data were analyzed using R software version 4.4.

**Results:**

A total of 327 neonates with suspected BSI were enrolled, with a median age of 1 day [interquartile range (IQR): 1–5 days]. The case-fatality rate was 9.2% (30/327). BSIs were confirmed in 53/327 (16.2%) neonates. Among confirmed cases, 39/53 (73.5%) were caused by Gram-negative bacteria, predominantly *Klebsiella pneumoniae* and *Acinetobacter* spp. Neonates with confirmed BSI had a significantly longer mean hospital stay than those with culture-negative sepsis (10.1 ± 8.0 days vs. 6.6 ± 6.6 days; *p* < 0.001). Elevated CRP was an independent predictor of BSI (*p* < 0.001).

**Conclusion:**

BSIs remain a significant cause of neonatal morbidity and mortality in this setting. Gram-negative organisms predominated and were associated with worse outcomes. A combination of clinical features, early warning signs, and CRP may aid in the early identification of neonates at high risk of BSIs and support timely initiation of appropriate management in resource limited settings.

## Introduction

Sepsis remains a major global health threat, with neonates bearing the highest burden of disease and mortality (1). The prevalence and mortality of neonatal bloodstream infections (BSI) are approximately twofold higher in low- and middle-income countries (LMICs) than in high-income countries (HICs) (2, 3). The World Health Organization (WHO), through Sustainable Development Goal (SDG) 3.2, has emphasized the need to improve neonatal survival, targeting a reduction in neonatal mortality to at least 12 per 1,000 live births by 2030 (4). Achieving this goal in LMICs would require substantial improvements in the timely recognition and appropriate management of BSI (5).

In Tanzania, the prevalence of laboratory-confirmed BSI among neonates varies widely, ranging from 11% at Bugando Medical Centre (BMC), Mwanza, to as high as 70% at Mwananyamala Hospital in Dar es Salaam (6, 7). At BMC specifically, the prevalence has increased from 11% in 2018 to 59% in 2022 among neonates admitted to the neonatal intensive care unit and nursery unit. Despite routine use of blood culture in these studies, a significant proportion of clinically suspected cases yield no identifiable pathogen (6, 8).

Furthermore, there is currently no standardized local guidelines to guide decisions on antibiotic initiation. A recent study by Khan N (9) reported that strict adherence to national and international guidelines may lead to a doubling of antibiotic prescriptions, highlighting the urgent need for context-specific, practical diagnostic tools (9). The etiological spectrum of neonatal BSI varies globally, with multidrug-resistant Gram-negative bacteria being predominant in Tanzania and other LMIC neonatal units. In particular, *Klebsiella pneumoniae* is frequently reported as the leading pathogen, posing significant challenges to effective treatment. Although blood culture remains the gold standard for BSI diagnosis, it is often unavailable in many LMIC settings and requires 48–96 h to yield results, potentially delaying appropriate management (10–12). In HICs, predictive models incorporating perinatal risk factors, vital signs, and laboratory biomarkers have been developed to facilitate early initiation of appropriate empirical therapy (13, 14). Biomarkers such as elevated C-reactive protein (CRP) and abnormalities in complete blood count (CBC) have been shown to predict both early and late onset neonatal sepsis in Tanzania and other settings (15, 16). However, readily measurable clinical parameters including blood glucose, temperature, oxygen saturation, respiratory rate, and heart rate remain underutilized in neonatal care in Tanzania. Importantly, abnormalities in these vital signs have been shown to identify neonates at high risk of clinical deterioration and may support timely and targeted management (6, 15, 17–21).

C-reactive protein (CRP) and complete blood count (CBC) are technically simple, cost-effective, and have a short turnaround time, and they do not require advanced laboratory infrastructure or highly specialized personnel. In this study, we conducted a comprehensive analysis of bloodstream infections (BSI) at the Department of Pediatrics and Child Health, Bugando Medical Centre (BMC), a major healthcare provider in the Lake Victoria region of Tanzania.

## Methodology

### Study design, area and duration

This was a hospital-based cross-sectional study conducted at Bugando Medical Centre (BMC), Tanzania, within the Department of Pediatrics and Child Health, specifically in the neonatal units. BMC is a zonal referral and teaching hospital affiliated with the Catholic University of Health and Allied Sciences, with a bed capacity of approximately 950. It serves the Lake Zone, with an estimated population of 16 million people from the regions of Kagera, Geita, Mwanza, Shinyanga, Simiyu, and Mara. The neonatal unit is divided into the neonatal intensive care unit (NICU) and the special care nursery. The NICU admits approximately five neonates per week, while the special care nursery receives an average of 17 admissions per week.

### Study population

The study included neonates admitted from 15th January 2023 to 30th June 2024 with clinical signs of sepsis from birth to 5 days of life. The clinical signs and symptoms included any of the following: temperature instability (hypothermia and fever), irritability, lethargy, respiratory symptoms (e.g., tachypnea, grunting, hypoxia), poor feeding, tachycardia, convulsion, jaundice, poor perfusion, and hypotension (22).

### Definitions

Bloodstream infection (BSI) was defined as the presence of clinical signs of sepsis accompanied by isolation of a causative bacterial pathogen in blood culture. Body temperature was measured in degrees Celsius using a digital thermometer (Tempo Max, Spengler, France). Hypothermia was defined as an axillary temperature <36.5°C on admission, while fever was defined as a temperature >37.5°C. Oxygen saturation (SpO₂) was measured using a pulse oximeter (UT 100, Germany), and hypoxia was defined as an oxygen saturation of <90% in both term and preterm neonates. Random blood glucose was measured using a haemoglucometer (Accu-Chek® Instant Blood Glucose Meter, Roche® GmbH, Germany). Hypoglycemia was defined as a blood glucose level <2.5 mmol/L, while hyperglycemia was defined as >8.3 mmol/L. Heart rate was assessed using a pulse oximeter (UT 100, Germany), with a normal range defined as 100–160 beats per minute. Respiratory rate was determined by counting breaths per minute, with a normal range of 40–60 breaths per minute. Preterm infants were defined as neonates born before 37 completed weeks of gestation (21).

Coagulase-negative staphylococcal (CoNS) infection was defined as the presence of a single positive blood culture for CoNS in conjunction with evidence of systemic inflammation, indicated by an elevated C-reactive protein (CRP ≥10 mg/L) and/or an abnormal white blood cell (WBC) as previously described (23, 24).

### Sample size and sampling procedure

A minimum sample size of 295 neonates with suspected BSIs was calculated using the Kish–Leslie formula (25). An estimated prevalence of 25.9% for confirmed BSI, derived from the average of previous studies conducted at Bugando Medical Centre, Tanzania, was used in the calculation (11, 20).

### Data collection procedure

#### Patient recruitment and specimen collection

Patient were screened by pediatricians (principal investigator and research assistants) using the guidelines published by the WHO Young Infants Study group (22) that require assessment of clinical signs and symptoms relevant to BSIs such as fever, convulsions, difficulty in breathing, jaundice etc.

Patients were screened and enrolled within five days of life (at admission, at birth or when developing signs and symptoms of BSI). Clinical information such as history of premature delivery, place of delivery, mode of delivery, birth weight, birth weight, vitals etc. were collected during enrollment. Additionally, physical examinations were done to all included neonates at the time of enrollment. In addition, blood samples were collected for CRP, CBC and culture.

Data was collected through interviews with caregivers and information on the child progress during hospitalization was obtained from medical charts. Following blood sample collection, appropriate treatments following the Tanzania neonatal guideline were initiated using ampicillin and gentamicin as first line, cefotaxime as second line and meropenem as third line (21) and the neonates were followed up until discharge or death.

#### Clinical samples and laboratory procedures

Approximately 3 mL of venous blood was collected from all neonates with clinical sepsis at enrollment using plain and Ethylenediaminetetraacetic acid (EDTA) BD Vacutainer tubes (BD Vacutainer, Nairobi, Kenya) for the determination of C-reactive protein (CRP) and complete blood count (CBC), respectively, as previously described (16, 26). The turnaround time for these tests was approximately two hours. Laboratory parameters were interpreted according to established criteria (27). Serial measurements of CRP and CBC were performed at admission, and at 24 and 72 h post-admission.

For blood culture, approximately 1 mL of venous blood was inoculated into 10 mL of in-house prepared tryptone soy broth (TSB) and incubated aerobically at 37°C for 18–24 h upon receipt in the laboratory. Blind subcultures were performed onto in-house prepared 5% sheep blood agar (SBA), MacConkey agar (MCA), and MacConkey agar supplemented with cefotaxime (30 µg/mL) (Oxoid, UK), followed by aerobic incubation at 37°C for 18–24 h. If no growth was detected, broth cultures were re-incubated and sub-cultured at 48 h, 96 h, and finally on day 7.

Bacterial identification and antimicrobial susceptibility testing were performed as previously described (28, 29). Classification of isolates as true pathogens or contaminants was conducted using standardized criteria described by Murni et al. (24). Quality control procedures were strictly followed to ensure the sterility and reliability of culture media and laboratory processes.

#### Data management and analysis

Data analysis was performed using R software (version 4.4). Descriptive statistics, including frequencies and proportions, were used to summarize demographic, maternal, and clinical characteristics of the study participants. To compare mean values of vital signs and hematological indices between neonates with and without sepsis, data normality was assessed using the Shapiro–Wilk test. As the normality assumption was not met, the Wilcoxon rank-sum test was applied for comparisons between groups. Univariate analysis was conducted using the Chi-square test or Fisher's exact test, as appropriate, to identify factors associated with bloodstream infection (BSI). Variables with potential associations were further included in a multivariate analysis to assess independent predictors while accounting for confounding factors. Statistical significance was set at *p* < 0.05.

#### Ethical consideration

The study got ethical clearance from the Joint Bugando Medical Centre/Catholic University of Health and Allied Sciences ethics and review committee with certificate no CREC/586/2022 and from the National Health Research Ethics Review Committee (NatHREC) with certificate number protocol NIMR/HQ/R.8a/Vol 1X/4318. Permission to conduct the study was granted by the BMC hospital authority. Written informed consent was sought from all parents/guardians of the neonates after full disclosure of the protocol. All neonates who had sepsis during the study were managed according to the BMC BSI protocol.

## Results

### Social demographic and clinical characteristics of the enrolled participants

A total of 327 (31.0%) neonates with clinical signs of bloodstream infection (BSI) were admitted between January 2023 and June 2024 out of 1,056 total neonatal admissions. Of these, 155 (47.4%) were male. The median age at admission was 1 day [interquartile range (IQR): 1–5 days]. Among the neonates, 138 (42.2%) were preterm. Clinical characteristics at admission included inability to breastfeed in 28 (8.6%) neonates, convulsions in 20 (6.1%), fever (temperature >37.5°C) in 30 (9.1%), and oxygen saturation <90% in 50 (15.2%). Regarding maternal factors, the majority of mothers (222/327, 67.9%) reported antibiotic use during the last month of pregnancy. Prolonged premature rupture of membranes (PROM ≥18 h) occurred in 30 (9.2%) cases, and 60 (18.3%) mothers had meconium-stained amniotic fluid during labor (Table 1).

**Table 1:** Social demographic and clinical characteristics of the 327 neonates with clinical sepsis admitted at bugando medical center, mwanza, Tanzania from January 2023 to June 2024^a^.

Variable	All N (%)	Pat. with BSI N (%)	Pat. without BSI N (%)	*P*
Sex				
Female	155 (47.4)	23 (14.8)	132 (85.2)	0.63
Male	172 (52.6)	30 (17.4)	142 (82.6)	
Age (days)	1.54 (0.98)			
Gestational Age				
≤36 6/7 weeks	138 (42.2)	23 (16.7)	115 (83.3)	0.97
≥37 weeks	189 (57.8)	30 (15.9)	159 (84.1)	
Birth weight				
≥ 2,500gm	203 (61.9)	30 (14.8)	94 (46.3)	
1,500 to 2,500gm	107 (32.6)	19 (17.8)	88 (82.2)	0.47
1,000 to 1,499gm	17 (5.5)	4 (23.5)	13 (76.5)	
Clinical symptoms				
Delayed crying	54 (16.5)	7 (13.0)	47 (87.0)	0.55
Difficulty in breathing	233 (71.3)	43 (18.5)	190 (82.6)	0.46
Irritability	12 (3.7)	3 (25)	9 (75)	0.42
Jaundice	49 (14.9)	6 (12.2)	43 (87.8)	0.53
Cyanosis	48 (14.7)	9 (18.8)	39 (81.2)	0.67
Inability to breastfeed	28 (8.6)	4 (14.3)	24 (85.7)	0.98
Convulsions	20 (6.1)	3 (15)	17 (85)	1.0
Axilla temperature				
≤36.5°C	104 (31.8)	20 (19.2)	84 (80.8)	
≥37.5°C	30 (9.1)	2 (6.7)	28 (93.3)	0.4202
36.5°C to 37.5°C	193 (59.1)	31 (16.1)	162 (83.9)	
Oxygen Saturation				
≥90%	277 (84.8)	43	234	0.5605
<90%	50 (15.2)	10	40	
History of fever during pregnancy				
Yes	55 (16.8)	10 (18.2)	45 (81.2%)	0.16
Maternal intrapartum fever				
Yes	9 (2.8)	2 (22.2)	7 (77.8)	0.8143
Antibiotic usage during pregnancy				
Yes	105 (32.1)	85 (81.0)	20 (19.0)	0.2820
Intrapartum antibiotics usage				
Yes	131 (40.1)	24 (18.3)	107 (81.7)	0.4251
Duration of rupture of the membrane				
Less than 18 h	297 (90.8)	50	247	0.4410
More than 18 h	30 (9.2)	3	27	
Meconium-stained liquor				
Yes	60 (18.3)	13 (21.7)	47 (78.3)	0.2820
Mode of delivery				
SVD^b^	141 (56.9)	117 (83)	24 (17)	0.8446
C/S^c^	186(43.1)	157(84.4)	29(15.6)	

a Table 1 describe the baseline characteristics of neonates with and without bloodstream infection (BSI). Values are presented as number and percentage, *N* (%) for categorical variables and mean (± standard deviation) for continuous variables. Comparisons between neonates with and without bloodstream infection were performed using the Chi-square test for categorical variables with expected cell counts ≥5 and the Fisher's exact test for variables with small expected counts (< 5), including maternal intrapartum fever and duration of rupture of membranes >18 h. A *p* < 0.05 was considered statistically significant.

b SVD, Spontaneous Vertex delivery;.

c C/S, Caesarian Section.

**Figure 1:**
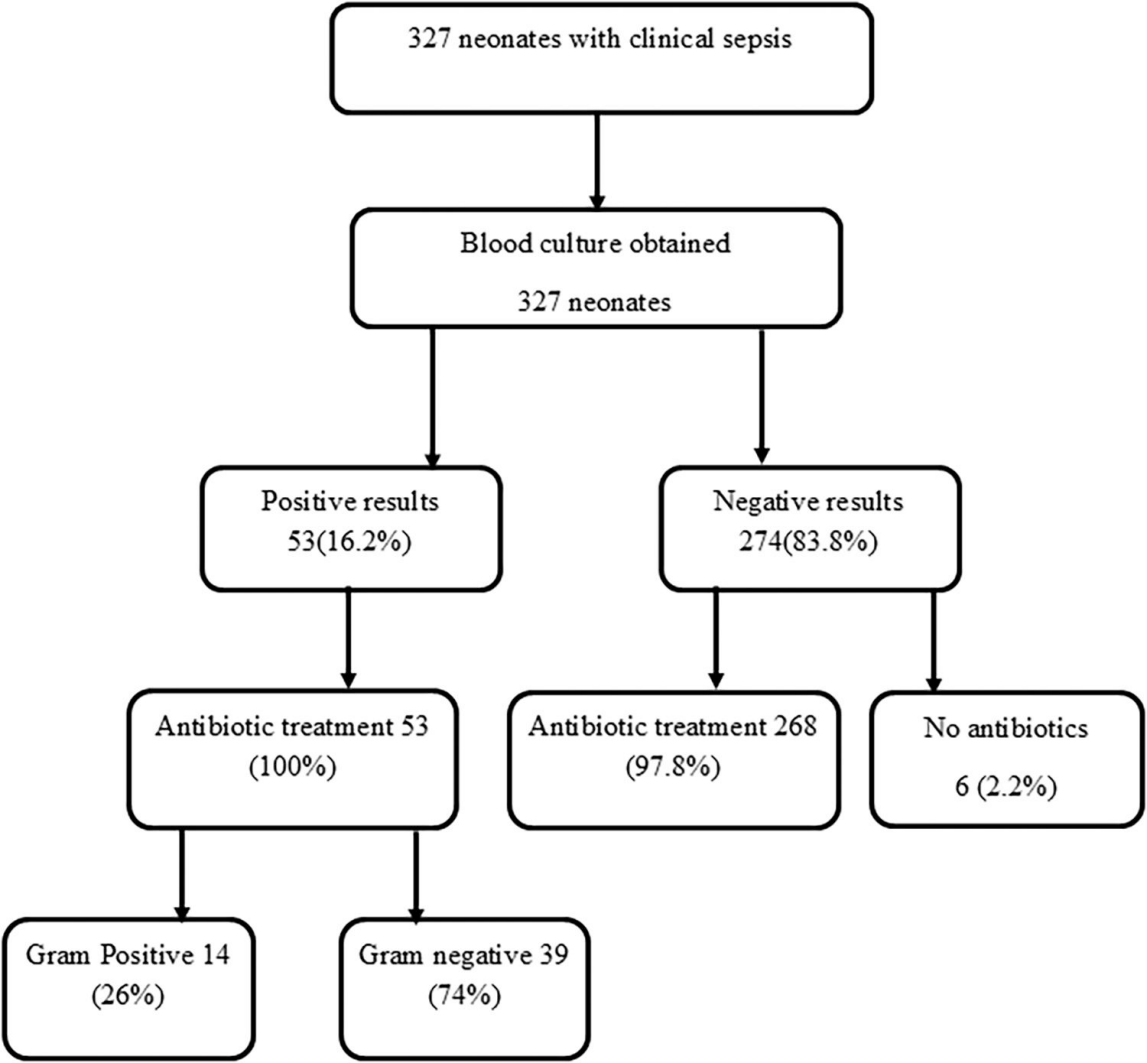
A flow chart shows 327 neonates with clinical sepsis and 53(16.2%) had positive culture results. Antibiotic treatment was initiated in 268(97.8%) neonates with negative culture results.

Among the 274 neonates with culture-negative sepsis, the majority (268, 97.8%) received antibiotic treatment. Overall, antibiotics were initiated at the time of enrollment in 321 (98.2%) of all enrolled neonates. The most commonly prescribed regimen was a combination of ampicillin and gentamicin (269, 83.8%), followed by cefotaxime (52, 16.2%) (Figure 1).

### Factors associated with bloodstream infections

Table 2 summarizes the admission vital signs (temperature, heart rate, respiratory rate, and random blood glucose) stratified by bloodstream infection (BSI). Overall, there were no significant differences in vital signs between neonates with culture-confirmed BSI and those with negative blood culture results, except for oxygen saturation. Neonates with BSI had significantly lower oxygen saturation levels at admission compared to those without BSI (*p* = 0.021). In addition, there were significant differences in admission leukocyte count (*p* = 0.031), platelet counts (*p* = 0.004) and C-reactive protein (*p* < 0.001) levels between neonates with culture-confirmed bloodstream infection (BSI) and those without confirmed sepsis. Neonates with culture-positive BSI had higher CRP levels, lower leukocyte counts, and lower platelets than those without confirmed infection (Table 2). On multivariate analysis, only CRP remained independently associated with BSI (OR = 1.05, 95% CI: 1.03–1.08, *p* < 0.001).

**Table 2:** Comparing vital signs and hematological parameters of the neonates with and without BSI admitted at the neonatal unit in bugando medical center in mwanza, Tanzania.

Variable	BSI Mean (±SD)	No BSI Mean (±SD)	*P*-value
Temperature ( °C, mean/SD)	36.5 (0.73)	36.7 (0.74)	0.38
Heart Rate (bpm, mean/SD)	140.7 (13.39)	143.9 (15.21)	0.4066
Respiratory Rate (breath, mean/SD)	59.7 (13.32)	59.8 (13.85)	0.9999
Random Blood Glucose (mmol/L, mean/SD)	5.0 (1.78)	5.0 (2.40)	0.4888
SPO_2_(%, mean/SD)	92.6849 (5.28)	94.4340 (5.85)	**0**.**0209**
Hemoglobin level (g/dL)	16.5	16.7	0.444
Monocyte (x10^9^/L	1.2	1.5	0.144
Lymphocyte (x10^9^/L)	3.7368	3.5922	0.678
Leucocyte (x10^9^/L)	12.5664	14.4127	**0**.**031**
Neutrophils (x10^9^/L)	7.6242	8.9912	0.068
Platelets (x10^9^/L)	191.4200	230.2422	**0**.**004**
C Reactive Protein (mg/L)	45.5924	9.3605	<**0**.**001**
Neutrophil to Lymphocyte ratio (NLR)	3.2	3.3	0.058

### Gram-negative pathogens dominate the pathogenic spectrum of BSI

Of the 327 neonates with clinical signs of sepsis, positive blood culture was found in 53 (16.1%, 95% CI: 12.0–20.0). Most of the isolated bacteria were Gram-negative (39/53, 73.6%). *Klebsiella pneumoniae, Coagulase-negative Staphylococcus (*CoNS), and *Acinetobacter* spp*.* were the most frequently isolated bacteria (Table 3).

**Table 3:** Distribution of bacterial organisms isolated from neonates with BSI.

Organism	Frequency	Percent
Gram-positive Cocci		
CoNS	11	20.8%
*Staphylococcus aureus*	2	3.8%
*Streptococcus spp.*	1	1.9%
Total	14	26.4%
Gram-negative Bacilli—Enterobacteriaceae		
*Escherichia coli*	2	3.8%
*Klebsiella pneumoniae*	25	47.2%
*Enterobacter aerogenes*	2	3.8%
Total	29	54.7%
Gram-negative Bacilli non-fermenters		
Acine*tobacter* spp.	8	15.1%
*Pseudomonas aeruginosa*	1	1.9%
Unidentified Gram-negative bacteria	1	1.9%
Total	10	18.9%
Total	53	100.0%

### Outcome among neonates admitted at bugando medical centre

Regarding treatment outcomes, the overall mortality rate was 9.2% (30/327). The mean duration of hospital stay was significantly longer among neonates with culture-positive BSI than those with culture-negative results (10.1 ± 8.0 days vs. 6.6 ± 6.6 days, *p* = 0.0009). The majority of neonates (62.7%) were hospitalized for 1–6 days, while 5.3% (16/327) stayed for more than 21 days. Mortality was higher among neonates with culture-positive BSI (15.1%, 8/53) than those with culture-negative results (8.0%, 22/274), although this difference was not statistically significant (*p* = 0.06). Furthermore, no deaths were observed among neonates with Gram-positive BSI, whereas 20.5% (8/39) of those with Gram-negative BSI died.

## Discussion

Neonatal morbidity and mortality remain major public health challenges, contributing substantially to under-five mortality, particularly in low- and middle-income countries (LMICs). This study assessed the prevalence, as well as clinical and hematological predictors, of bloodstream infections (BSI) among neonates. The overall mortality was 9.2%, with culture-confirmed BSI identified in 16.1% of neonates. The majority of isolates (73.6%) were Gram-negative bacteria, and elevated C-reactive protein (CRP) was independently associated with BSI.

The proportion of BSI (16.1%) observed in this study was lower than that reported in previous studies conducted in the same setting (11, 15) and other parts of East Africa (30, 31) but higher than reports from high-income countries (32, 33). The relatively lower prevalence in our study may reflect improvements in neonatal care and the implementation of national neonatal care guidelines in Tanzania (34) which have strengthened case management at lower-level health facilities. However, the widespread use of pre-referral antibiotics, as recommended by WHO guidelines, and the relatively small blood volume (1 mL) collected for culture may have reduced the diagnostic yield, thereby underestimating the true burden of BSI (35).

In this study, *Klebsiella pneumoniae*, coagulase-negative staphylococci (CoNS), and Acinetobacter *spp*. were the most frequently isolated pathogens which is consistent with previous findings from similar settings (36). Gram-negative bacteria formed the majority of the isolates in our study as previously reported in the same setting (11, 15). *Klebsiella pneumoniae* was the leading pathogen, accounting for approximately half of culture-confirmed BSI cases, in line with reports from this setting and other LMIC settings (6, 37–39).

The persistence of *Klebsiella pneumoniae* in neonatal units may be attributed to its ability to form biofilms, survive in hospital environments, and acquire antimicrobial resistance (40). In many LMIC settings, recurrent clonal outbreaks have been linked to overcrowding, limited infection prevention and control (IPC) capacity, and inadequate microbiological and genomic surveillance (41, 42). To better understand transmission dynamics and circulating strains, whole-genome sequencing (WGS) of isolates from clinical and environmental sources is recommended to inform targeted IPC strategies.

Clinically, neonates with BSI did not differ significantly in most vital signs compared to those without BSI, except for lower oxygen saturation at admission. Hypoxemia may represent an early warning sign of clinical deterioration, as previously reported [13,29]. Subtle abnormalities in physiological parameters such as oxygen saturation, temperature, and blood glucose may precede overt clinical signs of sepsis (19). When combined with laboratory markers such as CRP and platelet count, these indicators can support early identification and prompt management of neonatal sepsis, particularly in resource-limited settings.

Consistent with prior studies (15, 43) neonates with BSI had a significantly longer hospital stay compared to those without BSI. Mortality was higher among neonates with culture-positive infections and was observed exclusively among those with Gram-negative BSI, highlighting the clinical severity associated with these pathogens. This study has several limitations. First, the small blood volume (1 mL) collected for culture may have reduced pathogen detection rates. Second, prior antibiotic exposure among referred neonates may have further lowered culture positivity. Third, routine screening for Group B Streptococcus (GBS) and assessment of additional inflammatory markers such as procalcitonin and interleukin-6 were not performed, which may have limited comprehensive evaluation of neonatal sepsis.

## Conclusion

Bloodstream infections remain a significant cause of neonatal morbidity and mortality in this setting. Elevated CRP was an independent predictor of BSI, while hypoxemia and thrombocytopenia were important clinical and laboratory findings associated with BSI. Gram-negative organisms, particularly *Klebsiella pneumoniae*, predominated and were associated with worse outcomes. In resource-limited settings, a combination of clinical features, early warning signs, and accessible laboratory markers such as CRP may aid in the early identification of neonates at high risk of BSI and support timely initiation of appropriate management.

## Data Availability

The original contributions presented in the study are included in the article/Supplementary Material, further inquiries can be directed to the corresponding author/s.
